# Quality of English‐ and Spanish‐language online content about prostate cancer genetics: Insights into potential contributors to prostate cancer disparities

**DOI:** 10.1002/bco2.70011

**Published:** 2025-03-23

**Authors:** Sophia M. Abusamra, Verónica Ochoa Cholán, Veda N. Giri, Susan T. Vadaparampil, Verónica Pérez‐Rosas, Adrian Rivera, Tatiana Sanchez Nolasco, Mariana Rangel Camacho, Nataliya Byrne, Stacy Loeb

**Affiliations:** ^1^ Nuffield Department of Surgical Sciences University of Oxford Oxford UK; ^2^ Department of Urology and Population Health NYU Langone Health New York NY USA; ^3^ Department of Internal Medicine, Section of Medical Oncology Yale School of Medicine and Yale Cancer Center New Haven CT USA; ^4^ Department of Health Outcomes and Behavior Moffitt Cancer Center Tampa FL USA; ^5^ Department of Computer Science Texas State University San Marcos TX USA

**Keywords:** disparities, genetics, online content, prostate cancer, Spanish

1

Genetic testing is increasingly important for prostate cancer (PCa) care and the risk of hereditary cancer for patients and families.^1^ However, it is currently underutilized, notably among racial and ethnic minorities. In particular, prior studies have shown lower uptake of genetic evaluation among Hispanic patients with prostate cancer in the U.S. as well as those who are non‐English preferring, compared to those who are non‐Hispanic White and English‐preferring.^2^, ^3^

Health communications are important to raise knowledge and awareness about health issues and to increase demand for health services.^4^ The majority of U.S. adults go online for health information, and rates of social media use are particularly high among Hispanic adults.^5^ Our objective was to examine the extent and quality of online information about prostate cancer genetic testing and *BRCA* in English and Spanish. We hypothesized that there is less high‐quality online content about PCa genetics in Spanish than in English, as a potential contributor to the observed disparities in genetic evaluation.

We created a dataset with the first 25 websites listed on Google and the first 25 videos on YouTube (the most widely used social media platform^5^) with two different search terms (prostate cancer AND *BRCA*, prostate cancer AND genetic testing) in English and Spanish. These searches were selected based on an examination of Google trends data related to PCa genetics. We examined the first 25 websites and 25 videos using each of the two queries above, for a total of 50 websites in English, 50 websites in Spanish, 50 YouTube videos in English and 50 YouTube videos in Spanish. Videos were excluded if they were not in the correct language (English or Spanish), were not consumer health information (e.g., course for doctors) or >30 minutes in duration. Three investigators with clinical and/or research expertise in PCa independently examined the remaining relevant content from each platform using the validated DISCERN framework for the quality of consumer health information,^6^ which has been extensively used to evaluate websites and YouTube videos in English and Spanish.^7^, ^8^ We also examined understandability and actionability using the validated AHRQ Patient Education Materials Assessment Tool (PEMAT).^9^

We used descriptive statistics to tally the total number of relevant consumer videos using each search string, as well as their quality, understandability and actionability. Chi‐square and Mann–Whitney U tests were used to compare the proportion of relevant content and DISCERN and PEMAT scores, between Spanish and English content. We also created a composite measure for relevant videos that met quality criteria (DISCERN score of 4 or 5 out of 5, and both PEMAT scores >75% out of 100%).

Table 1 summarizes the results. Overall, 69% of English‐language videos and websites were relevant, compared to 51% Spanish‐language content (p = 0.02) [Table 1]. However, there was no significant difference in quality between English and Spanish content (mean DISCERN Q16 “Overall rating” score 3.1 vs 3.3, respectively, p = 0.52). The mean PEMAT scores for understandability (English: 61.9%, Spanish: 67.6%; p = 0.03) and actionability (English: 34.4%, Spanish: 48.5%; p = 0.02) were significantly higher for relevant Spanish content as compared to English content [Table 1]. Low mean PEMAT actionability scores for content in both languages indicate that the content lacked actionable instructions for lay health consumers to follow. Out of all 100 videos and websites, 23 in English and 21 in Spanish were relevant with a DISCERN quality rating of 4–5 (p = 0.86). Considering both DISCERN quality 4–5 and PEMAT understandability and actionability scores >75%, only 6 English and 12 Spanish websites/videos met the composite quality criteria (p = 0.15).

**TABLE 1:** Quality, understandability and actionability scores for websites and videos about prostate cancer genetics in English‐ and Spanish‐language (n = 120).

	English Google websites and YouTube videos					Spanish Google websites and YouTube videos					p‐value
	*PCa and BRCA Google (n = 25)*	*PCa and BRCA YouTube (n = 13)*	*PCa and genetics Google (n = 20)*	*PCa and genetics YouTube (n = 11)*	*Total (n = 69)*	*PCa and BRCA Google (n = 17)*	*PCa and BRCA YouTube (n = 7)*	*PCa and genetics Google (n = 20)*	*PCa and genetics YouTube (n = 7)*	*Total (n = 51)*	
Overall DISCERN rating*	3.4 (3.0)	2.8 (3.0)	3.0 (3.0)	3.0 (3.0)	3.1 (3.0)	3.7 (3.0)	3.0 (3.0)	3.1 (3.0)	3.0 (2.0)	3.3 (3.0)	0.52
PEMAT Understandability score**	60.0% (61.5%)	63.3% (61.5%)	66.5% (69.2%)	56.6% (53.8%)	61.9% (61.5%)	67.9% (76.9%)	68.1% (61.5%)	66.5% (73.1%)	69.2% (69.2%)	67.6% (69.2%)	0.03
PEMAT Actionability score**	24.0% (0.0%)	40.4% (50.0%)	47.5% (75.0%)	27.3% (25.0%)	34.4% (25.0%)	50.0% (47.1%)	53.6% (50.0%)	50.0% (50.0%)	50.0% (42.9%)	48.5% (50.0%)	0.02
**DISCERN 4 or 5 and relevant**†	9 (36.0%)	4 (30.8%)	7 (35.0%)	3 (27.3%)	23/100 (23.0%)	9 (52.9%)	3 (42.9%)	6 (30.0%)	3 (42.9%)	21/100 (21.0%)	0.86
Composite for good content*** ^**,**^ †	0 (0.0%)	2 (15.4%)	3 (15.0%)	1 (9.1%)	6/100 (6.0%)	6 (35.3%)	2 (28.6%)	2 (10.0%)	2 (28.6%)	12/100 (12.0%)	0.15

All data is presented as mean (median) unless otherwise noted. *1 is low, 3 is moderate, 5 is high, **Higher percentage is better for both PEMAT subscales, *** DISCERN 4/5, both PEMAT scores 75% or above and relevant, †*Data presented as N (%)*.

Statistical analyses revealed no significant correlations between scientific quality and viewer engagement, as measured by views per month and overall DISCERN (r = 0.22, p = 0.57), PEMAT Understandability (r = − 0.23, p = 0.45) or PEMAT Actionability (r = 0.33, p = 0.27) for YouTube videos in Spanish. Interestingly, YouTube videos resulting from a “PCa and *BRCA*” search had higher engagement (mean 83.7 views/month) than videos found with “PCa and Genetics” (mean 47.1). This could potentially reflect a broader public knowledge or interest specifically in “*BRCA*.”

We herein report a novel dataset used to study the dissemination of English‐ and Spanish‐language content about PCa genetics and *BRCA* on Google and YouTube. We hypothesized that less availability of high‐quality Spanish‐language content may serve as a contributor to overall lower uptake of genetic evaluation by Hispanic patients. Indeed, our results showed it is more difficult to find relevant Spanish‐language content on PCa and genetics or *BRCA* as compared to English. This corroborates prior work from Vanderpool and colleagues who surveyed Spanish‐only speakers who sought cancer information, reporting that their search took more effort, and was more frustrating compared to English‐speaking respondents.^10^

However, among the more limited available Spanish‐language content, there was similar quality with higher mean understandability and actionability as compared to relevant English‐language websites and videos. While this is encouraging, still there was very limited content that met all quality metrics. This points to the clear need for more attention to helping lay health consumers navigate the online information ecosystem.

Furthermore, our findings suggesting no correlation between the quality of information and viewer engagement highlight that popularity in online networks does not reflect better information. Thus, it is crucial for providers to direct patients to vetted materials for self‐education and for lay health consumers to identify content from trustworthy sources.

Additionally, only 3.9% of the content in Spanish had evidence of cultural tailoring. This could reflect an inability of content to relate to Hispanic patients through cultural factors, which potentially could negatively impact their subsequent uptake of genetic evaluation. To this end, Victorson et al. have previously described the importance of cultural competence for eHealth research tools with Latinx patient populations, highlighting cultural and linguistic factors that should be considered during the design and implementation of eHealth interventions with this population.^11^

Overall, these data indicate that there is less relevant Spanish‐language content on YouTube and Google pertaining to PCa genetics and *BRCA*. This could increase barriers to awareness and education for Spanish‐preferring patients, and thus impact uptake of genetic evaluation. Given the downstream harms associated with underutilization of germline testing (e.g., precluding access to precision therapeutic options and clinical trials, higher rates of variants of uncertain significance in minority populations due to limited genetic data, etc.), more work is needed to explore potential interventions to increase guideline‐concordant uptake of germline evaluation among Hispanic and Spanish‐preferring patients with PCa.

## CONFLICT OF INTEREST STATEMENT

SL reports consulting with Astellas, Savour Health, Endo Pharmaceuticals, Blue Earth and Doceree, and research funding from Endo Pharmaceuticals, unrelated to the current study.

## ACKNOWLEDGMENTS

This study was supported by a grant from the Department of Defense (HT94252311040).
